# 321 Paired associative stimulation: a tool for assessing sensorimotor neural signaling and lower limb function post-stroke – ADDENDUM

**DOI:** 10.1017/cts.2023.563

**Published:** 2023-06-14

**Authors:** Jasmine Cash, Kirstin-Friederike Heise, John Kindred, Mark Bowden

The above abstract [1] published without listing Kirstin-Friederike Heise as an author.

The original abstract has been corrected online to rectify this omission.
